# Characteristics, Domestication and Nutrient Analysis of Wild *Pleurotus placentodes*

**DOI:** 10.3390/foods14223975

**Published:** 2025-11-20

**Authors:** Linna Cai, Xiaomin Li, Xin Hu, Junli Zhang, Huijuan Sun, Lei Gao, Junsheng Fu

**Affiliations:** 1 College of Life Sciences, Fujian Agriculture and Forestry University, Fuzhou 350002, China; 13297338949@163.com (L.C.); lxm2651277345@163.com (X.L.); hx1962024@163.com (X.H.); 2 Tibet Academy of Agricultural and Animal Husbandry Sciences, Lhasa 850002, China; xzyyxh888@163.com (J.Z.); m18898002012@163.com (H.S.); gaoleilei3@163.com (L.G.)

**Keywords:** wild mushroom, *Pleurotus placentodes*, cultivation, domestication, nutritional value

## Abstract

To enhance the systematicity and integrity of mushroom resource research by supplementing the mushroom germplasm bank through the collection of wild mushroom specimens, this project focused on a wild mushroom strain collected from Tibet Autonomous Region. The strain was identified and characterized through morphological observation, ITS sequencing, biological trait analysis, and cultivation experiments. The results indicated that the strain belongs to *Pleurotus placentodes*. The optimal growth conditions for this strain were determined as follows: fructose and sucrose as the preferred carbon source, yeast powder and beef powder as the optimal nitrogen source, pH 5.0, and a temperature of 20 °C. In cultivation experiments, the mycelium fully colonized the substrate in 23 days, primordia formation occurred within 10 d, and the fruiting period lasted 7 d, with an average fresh weight of 39.40 g per fruiting body. Nutritional analysis revealed that *P. placentodes* has a high protein content (30.30%), high soluble sugar content (6.60%), low fat content (1.00%), and a rich profile of essential amino acids (7.96%). This study successfully isolated, identified, and conducted domestication cultivation and nutritional analysis of a wild *P. placentodes*, providing scientific references for its potential industrial-scale production.

## 1. Introduction

*Pleurotus* mushrooms are a type of edible mushroom with great medicinal potential and edible value. These mushrooms contain diverse bioactive compounds including terpenoids, steroidal compounds, phenolic acid derivatives, and polyynes, with polysaccharides being particularly noteworthy. These bioactive constituents exhibit a wide range of pharmacological activities, such as antioxidant, anti-aging, anti-inflammatory, immunomodulatory, antitumor, anti-obesity, hypolipidemic, and hypoglycemic effects [1,2,3,4].

*Pleurotus placentodes* is a rare species of basidiomycetous fungus classified within the order Agaricales, family Pleurotaceae, and genus *Pleurotus*. *P. placentodes* was first discovered by the British mycologist Berkeley, M.J. in 1852 and published as a new species of the genus *Agaricus* [5]. Since then, there has been no new report on this species in the world for 164 years since its publication [6]. Until recently, researchers from the Kunming Institute of Botany, Chinese Academy of Sciences rediscovered it in the subalpine forests at an altitude of 3000–4200 m in Tibet and Yunnan, China, and proved that *P. placentodes* is mainly distributed in the eastern Himalayas–Hengduan Mountains. It is a rare edible mushroom unique to China [6].

Despite the recent rediscovery of *P. placentodes*, systematic research on its physiological growth responses and nutritional characteristics remains scarce. Previous studies have primarily focused on its taxonomic identification and ecological distribution, while comprehensive quantitative analyses on carbon/nitrogen utilization, pH and temperature optimization, yield performance, and proximate nutrient composition are still lacking. To address these research gaps, our team investigated a wild *P. placentodes* specimen collected in 2023 from the Sygera Mountains, Bomi County, Nyingchi City, Tibet Autonomous Region (94°716′ E, 29°651′ N; altitude 3850 m). The objectives of this study were to: (i) verify the morphological and molecular identification of the Tibetan strain X21214; (ii) quantitatively evaluate the effects of carbon, nitrogen, pH, and temperature on mycelial growth; (iii) assess the feasibility and yield performance of artificial domestication; and (iv) determine the proximate composition and amino acid profile of the fruiting bodies on a dry-weight basis. The comprehensive findings of this study provide a scientific foundation for the industrial cultivation and nutritional evaluation of *P. placentodes*.

## 2. Materials and Methods

### 2.1. Experimental Materials

#### 2.1.1. Wild Fruiting Bodies

The wild fruiting body sample was collected from the Sygera Mountains, Bomi County, Nyingchi City, Tibet Autonomous Region, China. The specimen is preserved at the Fungal Culture Center of Fujian Agriculture and Forestry University (accession No. X21214). The mycelium was obtained by tissue isolation from the fruiting body.

#### 2.1.2. Culture Medium

Enriched PDA medium: 200 g peeled potato, 20 g glucose, 5 g peptone, 2 g KH_2_PO_4_, 1.5 g MgSO_4_, 10 mg Vitamin B1 (V_B1_), 20 g agar, 1 L water, pH natural.

Liquid medium: 200 g peeled potato, 20 g fructose, 5 g peptone, 1.5 g MgSO_4_, 2 g KH_2_PO_4_, 10 mg V_B1_, 1 L water, pH natural.

Substrate for cultivation materials: 60% miscellaneous woodchips, 20% cottonseed hulls, 18% wheat bran,1% lime, and 1% white sugar were mixed in proportion (all proportions on a *w*/*w* dry-weight basis). The dry ingredients were thoroughly mixed, and water was added gradually while stirring until the substrate reached a moisture content of 62%, measured by oven-drying to constant weight [7].

### 2.2. Experimental Methods

#### 2.2.1. Identification of the Strain

The size, shape and color of the pileus, the morphology of the stipe, and the texture and color of the mycelium of the collected fruiting bodies were measured and recorded. Preliminary classification of the wild fungus was conducted with reference to *Atlas of Chinese Macrofungal Resources* [8] and *Illustrated Handbook of Chinese Macrofungi* [9].

Molecular identification: The pure DNA template was extracted using OMEGA fungal DNA extraction kit. Subsequently, the DNA was used as a template, and the universal fungal primers ITS1/ITS4 primer pair [7], which is widely used in the identification of mushroom in the ITS region, was used for PCR amplification according to the method described by Jin et al. [10]. The PCR program consisted of an initial denaturation at 95 °C for 5 min, followed by 35 cycles of 95 °C for 30 s, 58 °C for 30 s, and 72 °C for 90 s, and a final extension at 72 °C for 7 min. After PCR amplification, 3 μL PCR products were analyzed by 1% agarose gel electrophoresis to verify the quality and specificity of the amplified products. If the electrophoresis results show a single and bright band, it indicates that the amplification is successful, and then the remaining PCR products are entrusted to Fuzhou Baijing Biotechnology Co., Ltd. (Fuzhou, China) for sequencing services. The obtained sequences were accurately submitted to the NCBI nucleotide database (URL: http://www.ncbi.nlm.nih.gov/, accessed on 17 May 2025). Subsequently, the obtained ITS sequence was submitted to GenBank and assigned the accession number PQ094104 (Submission date: 31 July 2024). Using MEGA 12.0 software, the ITS sequences with high similarity were aligned with reference sequences from related *Pleurotus* species using the MUSCLE algorithm (default parameters). Poorly aligned or ambiguous regions were manually trimmed to ensure positional homology. Phylogenetic analysis was performed using the maximum likelihood (ML) method under the Kimura 2-parameter (K2P) substitution model. The ITS sequence of *Xylaria berteroi* was used as the outgroup for tree rooting and comparative analysis.

#### 2.2.2. Study on Growth Characteristics

The optimum carbon source factor test: Refer to Hu et al. [11] and other methods and slightly modified. Enriched PDA medium was used as the basal medium to study the effects of different carbon sources (as described in Section 2.1.2). The activated plate strains were taken and punched at the edge with a 5 mm diameter puncher. The fungus blocks were selected and placed in the center of a medium plate (9 cm) with different carbon sources (glucose, sucrose, fructose, maltose, mannose, starch), each at a concentration of 20 g/L and at 25 °C maintaining a constant temperature incubator. After 7 d of incubation, the colony diameter was measured by two-perpendicular-diameter method. Each treatment was replicated 5 times, and the growth indexes such as mycelial growth and density were observed. The mycelial growth rate (mm/day) was calculated as:

(1) $$Mycelial\;Growth\;Rate(mm/day)=\frac{\left[Colony\;Diameter\left(mm\right)\right]-[Inoculum\;Diameter\left(mm\right)]}{Growth\;Duration(d)}$$

The optimum nitrogen source factor test: enriched PDA medium was used as the basal medium to study the effects of different nitrogen sources. The specific operation steps were the same as the optimum carbon source factor test. The fungus blocks were inoculated into different nitrogen sources (urea, yeast powder, (NH_4_)_2_SO_4_, beef powder, peptone, NH_4_NO_3_) for culture, the concentration of each nitrogen source was 5 g/L. Each treatment was replicated 5 times.

The optimum pH test: enriched PDA medium was used for the pH optimization experiment. After the culture medium has been sterilized, adjust its pH using filter-sterilized 0.1 mol/L NaOH or HCl solution, and measure the pH with a pH meter. Gradient values of 5, 6, 7, 8, 9, and 10 were set. The procedure was the same as that used for the optimum carbon source test, with fungal blocks inoculated into media of different pH values (5, 6, 7, 8, 9, and 10) for cultivation. Each treatment was replicated 5 times.

The optimal temperature factor test: the enriched PDA medium was used as the basal medium for the temperature range experiment. The medium was incubated in constant-temperature chambers at 15, 20, 25, 30, 35, and 40 °C under dark conditions. Subsequent procedures were the same as those used in the carbon source experiment. Each treatment was replicated 5 times.

#### 2.2.3. Domestication Cultivation

The liquid medium was prepared as described in Section 2.1.2. Aliquots of 100 mL were dispensed into 250 mL Erlenmeyer flasks and sterilized by autoclaving. After cooling to room temperature, mycelial discs (5 mm diameter) were collected from culture plates and used to inoculate each flask with five discs. The flasks were sealed and incubated on a rotary shaker at 25 °C and 160 rpm in the dark for 7 d to produce liquid spawn.

Based on previous studies [12,13], which demonstrated successful growth of *P. placentodes* on substrates containing wood chips, cottonseed hull, and wheat bran, we adopted a similar substrate composition for domestication and cultivation.

The substrate was prepared according to the method in Section 2.1.2. Each cultivation bag was filled with 900 g (wet weight) of the substrate and equipped with a 0.2–0.5 micron filter membrane for gas exchange. It was sterilized using the standard autoclaving procedure that maintained at 121 °C for 2 h and then cooled to below 30 °C before removal. After cooling, each bag was aseptically inoculated with 20 mL of liquid spawn, which was equivalent to one—fifth of the spawn amount in a 100 mL spawn culture flask. The dry weight concentration of the mycelium in the liquid spawn used was approximately 8–10 mg/mL, which served as an indicator of biomass. Inoculated bags were incubated in darkness at 20–23 °C. Once the mycelium had fully colonized the substrate, bags were transferred to a fruiting room maintained at 18–20 °C and 95% humidity to induce primordia formation. The temperature was then raised to 20–23 °C to support fruiting body development. During cultivation, key growth and yield parameters were recorded, including time required for mycelial colonization, primordia initiation, and fruiting body maturation. The fresh and dry weights of harvested fruiting bodies from each bag were measured using an electronic balance. Biological efficiency (BE, %) was calculated as follows:

(2) $$BE\left(\%\right)=\frac{Fresh\;weight\;of\;fruiting\;bodies\left(g\right)}{Dry\;weight\;of\;substrate\left(g\right)}\times 100$$

#### 2.2.4. Analysis of Nutritional Value Components

Fresh fruiting bodies of *P. placentodes* were dried in a forced-air oven at 70 °C to constant weight. The dried samples were ground and passed through a 60-mesh sieve to obtain a homogeneous powder. For fat analysis, a separate portion of the powder was defatted by Soxhlet extraction with petroleum ether (6–8 h). All other chemical analyses were performed using the non-defatted powder.

Moisture content was measured at 70 °C to constant weight. Protein (Kjeldahl), ash (muffle furnace), fat (Soxhlet), total soluble sugars (HPLC-ELSD), dietary fiber (enzymatic-gravimetric), and sodium (flame atomic emission) were determined following AOAC (2010) methods [14]. Analytical conditions for soluble sugar (primarily mono- and disaccharides) quantification by HPLC-ELSD: amino-bonded column (250 × 4.6 mm, 5 μm); mobile phase: acetonitrile-water (70:30, *v*/*v*); flow rate: 1.0 mL/min; column temperature: 40 °C; ELSD settings: drift tube temperature 85 °C, nitrogen pressure 350 kPa; quantification via external calibration with glucose, fructose, sucrose, maltose, and lactose standards (0–10 mg/mL). Results are expressed on a dry-weight basis (g/100 g dry matter) unless otherwise stated. For amino-acid analysis, samples were hydrolyzed with 6 mol/L HCl at 110 °C for 24 h under nitrogen. Tryptophan is destroyed under these conditions, and cysteine and methionine may partially degrade; therefore, only amino acids reliably quantified by acid hydrolysis are reported in Table 1, and values for tryptophan and cysteine are omitted.

### 2.3. Statistical Analysis

The experiments were conducted using a completely randomized design. Each treatment consisted of five biological replicates (n = 5). The data were processed using Microsoft Excel and subjected to statistical analysis with IBM SPSS Statistics 27.0. The significance of differences among treatment means was determined by one-way analysis of variance (ANOVA), followed by post hoc comparisons using the Least Significant Difference (LSD) test. The results are presented as the mean ± standard deviation. Differences were considered statistically significant at *p* < 0.05. Graphical representations of the data were generated using GraphPad Prism 9.0.

### 2.4. Main Reagents

Maltose, mannose, fructose, starch, sucrose, glucose, urea, yeast powder, (NH_4_)_2_SO_4_, beef powder, peptone, NH_4_NO_3_, KH_2_PO_4_, MgSO_4_, agar, and other reagents (analytical grade, purchased from China National Pharmaceutical Group Corporation (Beijing, China)). D3390-01 Fungal DNA Kit (OMEGA Bio-Tek, Norcross, GA, USA).

## 3. Results

### 3.1. Identification of Strain X21214

#### 3.1.1. Morphological Identification of Strains

**Figure 1:**
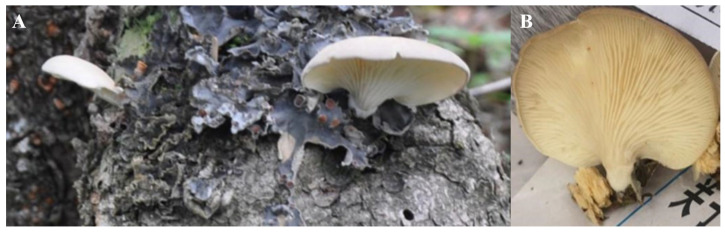
Wild fruiting bodies (**A**) and gills (**B**) of strain X21214.

The fruiting body morphology of X21214 wild samples is shown in Figure 1A,B. Pileus applanate to infundibuliform, cup-shaped or flabelliform-shaped, dark gray to pale yellowish-brown with a wavy, involute margin. Lamellae decurrent, unequal. Stipe clavate, lateral (rarely central), white to pale brown with longitudinal striations; base with short, ivory-white tomentum; contextual substance somewhat tough. The strain X21214 was preliminarily identified as *P. placentodes* based on morphollogicalcharacters described in *Atlas of Chinese Macrofungal Resources* [8] and *Illustrated Handbook of Chinese Macrofungi* [9].

#### 3.1.2. Molecular Identification of Strain X21214

**Figure 2:**
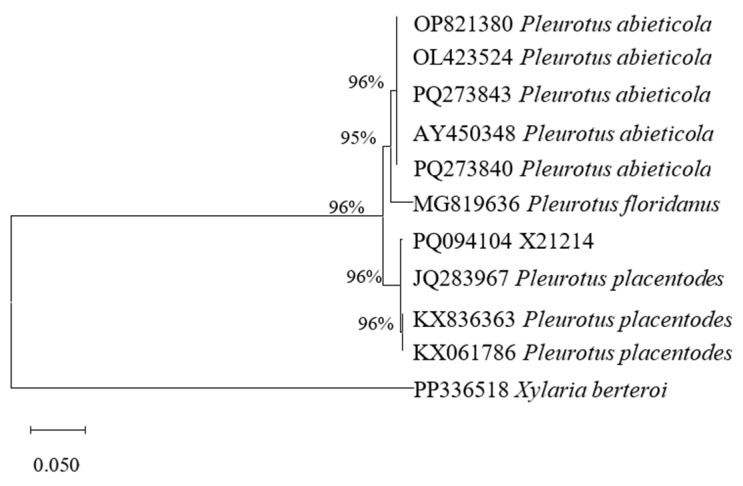
Maximum likelihood based on ITS sequences.

The ITS sequence length of strain X21214 was 639 bp and the GenBank accession number was PQ094104. By phylogenetic analysis, it was in the same branch with *P. placentodes* (Figure 2), and the homology reached 96%. Combined with the morphological identification results obtained in Section 3.1.1., the strain X21214 was supported as *P. placentodes.*

### 3.2. Growth Characteristics Experiment

#### 3.2.1. Growth of *P. placentodes* X21214 Mycelia on Different Carbon Sources

**Figure 3:**
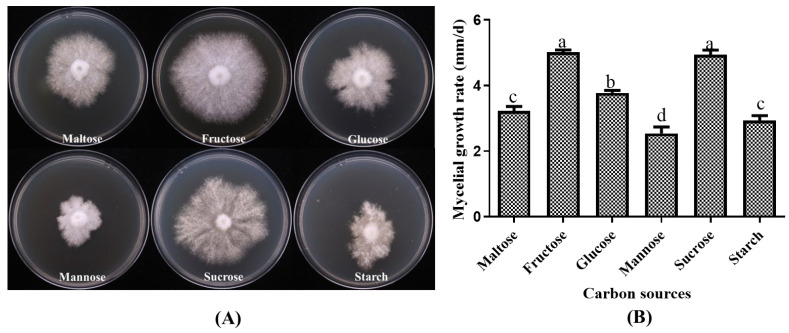
Effects of different carbon sources on mycelial growth of *P. placentodes* X21214. (**A**) Mycelial growth in various media. (**B**) Growth rate of mycelium under different media. Lowercase letters on the column chart indicate significant differences between different letters marked with the same index (*p* < 0.05).

Statistical analysis of the growth of the strain *P. placentodes* X21214 on different carbon sources revealed significant differences (Figure 3). The results showed that the optimum carbon sources for mycelial growth were fructose (5.01 ± 0.16 mm/d) and sucrose (4.94 ± 0.24 mm/d), followed by glucose (3.77 ± 0.13 mm/d) and maltose (3.22 ± 0.344 mm/d). In contrast, the strain exhibited poor growth on starch (2.93 ± 0.37 mm/d) and mannose (2.54 ± 0.47 mm/d), characterized by slow and weak mycelial development with irregular margins.

#### 3.2.2. Growth of *P. placentodes* X21214 Mycelia on Different Nitrogen Sources

**Figure 4:**
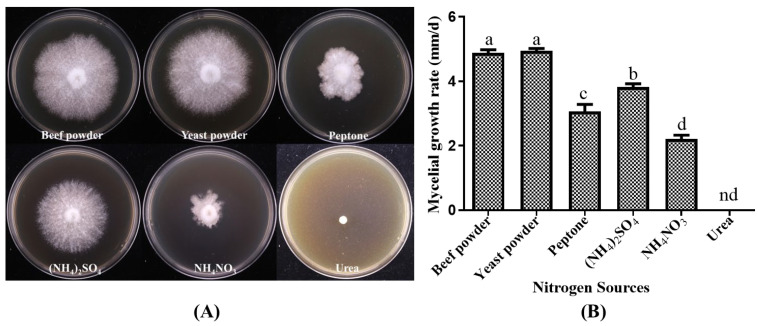
Effects of different nitrogen sources on mycelial growth of *P. placentodes* X21214. (**A**) Mycelial growth in various media. (**B**) Growth rate of mycelium under different media. Lowercase letters on the column chart indicate significant differences between different letters marked with the same index (*p* < 0.05), among them, "nd" indicates no growth.

The growth response of the strain *P. placentodes* X21214 to different nitrogen sources varied significantly (Figure 4). Yeast powder (4.94 ± 0.18 mm/d) and beef powder (4.88 ± 0.25 mm/d) were identified as the most favorable nitrogen sources, supporting robust mycelial growth with high density and vitality. Moderate growth was observed with (NH_4_)_2_SO_4_ (3.82 ± 0.26 mm/d and peptone (3.07 ± 0.52 mm/d), whereas NH_4_NO_3_ (2.21 ± 0.30 mm/d) supported only poor growth, and urea resulted in negligible mycelial development.

#### 3.2.3. Growth of *P. placentodes* X21214 Mycelia on Different pH

**Figure 5:**
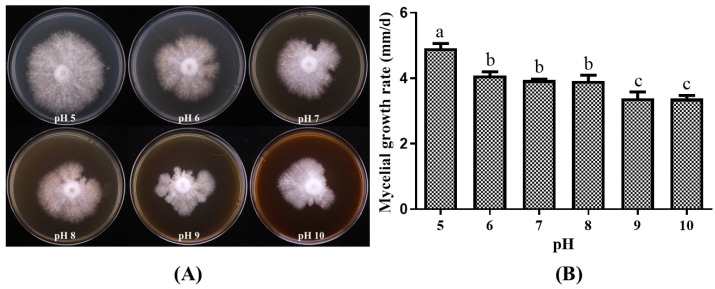
Effects of different pH on mycelial growth of *P. placentodes* X21214. (**A**) Mycelial growth in various media. (**B**) Growth rate of mycelium under different media. Lowercase letters on the column chart indicate significant differences between different letters marked with the same index (*p* < 0.05).

Variations in pH significantly affected the mycelial growth of *P. placentodes* X21214 (Figure 5). The growth rate decreased gradually as the pH increased. The fastest growth rate of 4.92 ± 0.36 mm/d was observed at pH 5, where the mycelia exhibited an optimal growth pattern on agar plates, characterized by dense, radial growth and high vitality. In contrast, the slowest growth rate of 3.38 ± 0.20 mm/d was recorded at pH 10.

#### 3.2.4. Growth of *P. placentodes* X21214 Mycelium on Different Temperatures

**Figure 6:**
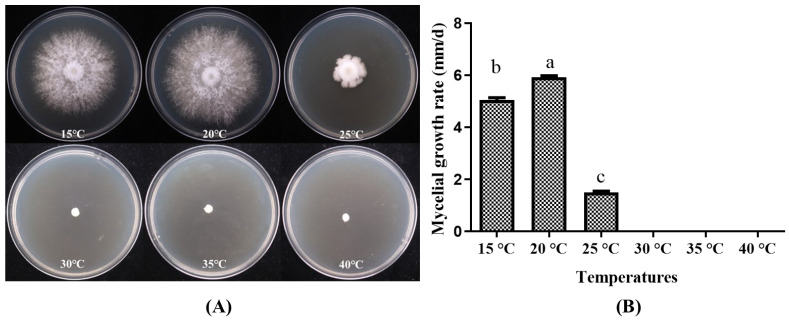
Effects of different temperatures on mycelial growth of *P. placentodes* X21214. (**A**) Mycelial growth in various media. (**B**) Growth rate of mycelium under different media. Lowercase letters on the column chart indicate significant differences between different letters marked with the same index (*p* < 0.05).

The strain *P. placentodes* X21214 growth on different temperature ranges found statistically significant (Figure 6) that the optimum temperature for mycelial growth recorded at 20 °C (5.92 ± 0.17 mm/d), followed by 15 °C (5.05 ± 0.19 mm/d). When the temperature is ≥30 °C, the mycelium does not germinate.

### 3.3. Domestication Cultivation and Agronomic Traits of Fruiting Body

The domestication cultivation results showed that after inoculation, the mycelium fully colonized the bags in approximately 23 d under dark conditions at 20–23 °C. 7 d after full colonization, the temperature was reduced to 18–20 °C and the relative humidity was maintained at approximately 95% to stimulate primordia differentiation through low-temperature induction. Primordia formed within 10 d. After primordia differentiation, the temperature was adjusted back to 20–23 °C, leading to mushroom emergence within 7 d, at which point harvesting could be conducted. The entire process from inoculation to fruiting required approximately 40 d.

**Figure 7:**
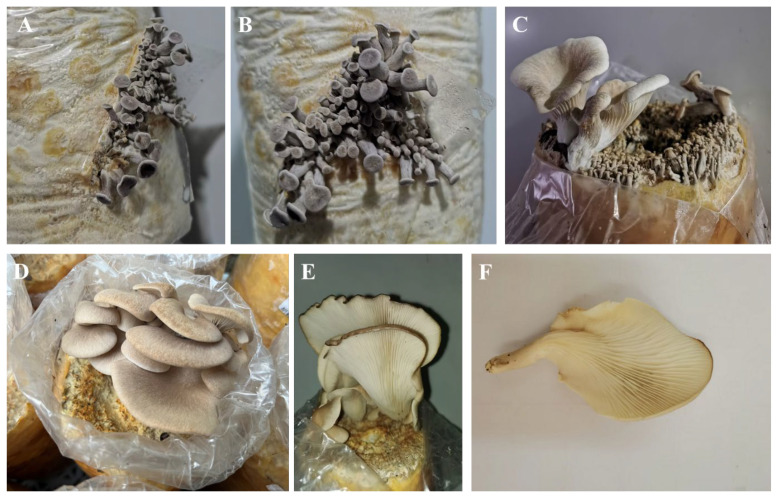
Primordia (**A**,**B**) and fruiting bodies (**C**–**F**) in the artificial cultivation process of *P. placentodes*.

The wet weight of each bag of substrate is 900 g, and there is approximately 342 g of dry matter. The first flush of fruiting bodies yielded an average fresh weight of 39.40 g per mushroom per bag, with a biological efficiency of 11.5%. The growth characteristics of the cultivated *P. placentodes* are shown in Figure 7. The mycelium was white, and the primordia initially appeared dark gray (Figure 7A,B). The pileus was discoid, flattened or slightly depressed, and tapered from the base to the margin. As the fruiting bodies developed, their color gradually shifted to creamy white (Figure 7C,D). During maturation, the central or subcentral area of the pileus became depressed, forming a cup-shaped or fan-like appearance. The center was light yellowish-brown, while the margin was grayish-brown with a wavy, curled edge. The stipe was lateral, covered with tomentum, and ivory-white in color, exhibiting slight toughness. The context was white and relatively thin, and the lamellae were straight and radiated outwards (Figure 7E,F).

### 3.4. Analysis of Nutritional

#### 3.4.1. Basic Nutrients

It can be seen from Table 2 that the water content of fresh *P. placentodes* was 91.4%, and the water content under dry weight was 16%. The nutrient content was measured in the dry weight state. The protein content of *P. placentodes* was 30.3%, and the soluble sugar content was 6.6%. The ash content of *P. placentodes* was 6.5%.

#### 3.4.2. Amino Acid Composition Analysis

The composition and proportion of amino acids, especially essential amino acids, are important indicators for evaluating the nutritional value of proteins. According to the analysis of the amino acid composition of fruiting bodies (Table 3), it was found that the fruiting bodies of *P. placentodes* were rich in non-essential amino acids and essential amino acids, including 16 kinds of amino acids, including 8 kinds of essential amino acids required by the human body.

## 4. Conclusions and Discussion

*P. placentodes* is a fungal species belonging to the family Pleurotaceae. It was first described by the British mycologist Berkeley from decaying birch (*Betula* spp.) wood in Sikkim, India [5].

The strain X21214 isolated in this study exhibited morphological features consistent with those of *P. placentodes* [8,9] and showed the highest ITS sequence similarity (96%) to the reference strain of *P. placentodes* (GenBank accession no. PQ0941041). Phylogenetic tree analysis further confirmed that strain X21214 clustered within the *P. placentodes* clade, indicating a close genetic relationship. Similar ITS-based identification approaches have been widely used for species delimitation in mushrooms, as the ITS region provides sufficient variability to distinguish closely related taxa [6,10,16,17]. According to Schoch et al. [18], an ITS sequence similarity above 99% is typically considered a robust threshold for species-level identification among Basidiomycetes. Our findings are consistent with those of Liu et al. [6], who rediscovered *P. placentodes* and confirmed its phylogenetic position within the *Pleurotus* lineage, and Wang et al. [13], who reported similar ITS homology in wild *P. placentodes* strains from Tibet. Therefore, both morphological and molecular evidence conclusively support the identification of strain X21214 as *P. placentodes*.

When cultivated on a substrate composed mainly of miscellaneous woodchips, cottonseed hulls, and wheat bran, *P. placentodes* showed strong adaptability and rapid mycelial colonization, suggesting high nutrient utilization efficiency and active lignocellulolytic enzyme systems [12,13]. The relatively short colonization period (approximately 23 days) and rapid primordia initiation under low-temperature induction (18–20 °C) indicate that *P. placentodes* is well adapted to cool environments, consistent with its natural occurrence in subalpine habitats [6]. This cold tolerance may be associated with elevated activities of cold-responsive enzymes and efficient carbohydrate metabolism, as reported for *P. ostreatus* and *Pleurotus pulmonarius* under similar temperature regimes [16,19].

The first-flush biological efficiency (11.5%) demonstrated that this species can be successfully domesticated, though further optimization of the substrate formulation—such as fine-tuning the carbon-to-nitrogen ratio or adding trace minerals—may improve yield, as shown in other *Pleurotus* cultivation systems [20,21]. Overall, these results confirm that *P. placentodes* is amenable to artificial cultivation and provide valuable technical references for its future commercial development.

The protein and soluble sugar content of *P. placentodes* were significantly higher than that of the three edible mushrooms (*P. eryngii*, *F. velutipes,* and *P. ostreatus*) [15]; the fat content of *P. placentodes* was 1.0%, which was significantly lower than that of the three edible mushrooms [15]. Dietary fiber refers to carbohydrates that cannot be decomposed by human digestive enzymes. The dietary fiber content of *P. placentodes* was 29.2%, which was lower than that of the three edible mushrooms [15]. The crude ash in wild edible mushroom is composed of inorganic salts and heavy metals, and its content can reflect the content of heavy metals in the soil where wild edible mushroom grow [22]. The ash content of *P. placentodes* was 6.5%, which was higher than that of *P. eryngii,* and lower than that of *F. velutipes* and *P. ostreatus*. The results showed that *P. placentodes* was a kind of edible fungus with high protein and low fat, rich in soluble sugar and minerals, which was beneficial to digestion and had high nutritional value.

The amino acids identified play diverse physiological roles [23,24,25,26,27,28,29,30,31]. The amino acids and essential amino acids contents of *P. placentodes* were 21.5% and 7.96%, respectively, which were higher than those of the three edible mushrooms (*P. eryngii*, *F. velutipes* and *P. ostreatus*) [15]. The ratio of the total essential amino acid content to the total amino acid content was 0.37. The contents of aspartic acid, serine, arginine, methionine, and proline were more than twice those found in the three comparison edible mushroom species. Furthermore, the levels of glycine, threonine, alanine, leucine, and lysine were slightly higher than those in *P. eryngii*, *F. velutipes*, and *P. ostreatus*. The combined presence of these amino acids suggests that *P. placentodes* possesses comprehensive nutritional and functional potential in metabolism regulation, tissue repair, and disease prevention, making it a valuable health-promoting edible mushroom.

At present, only limited studies have reported on the polysaccharides or secondary metabolites of *P. placentodes*. Existing research has primarily focused on its optimal growth conditions (temperature and pH) rather than detailed physiological or biochemical characterization. Morphological comparisons in this study revealed that domesticated fruiting bodies closely resembled their wild counterparts but exhibited larger size and better development.

Recent studies have also shown that Some *Pleurotus* species can biodegrade polyethylene terephthalate (PET) [32] and possesses biosynthetic potential [33]. Yin et al. [34] isolated and characterized a 3-*O*-methylated heteroglycan (PPp-W) from its fruiting bodies with notable anticoagulant and anti-inflammatory properties, while the ethanol extract (PP-a) demonstrated hepatoprotective activity [35]. These findings provide new perspectives for the development and utilization of *P. placentodes* in functional foods and medicinal applications.

In summary, this study achieved the isolation, molecular identification, and successful domestication of a wild *P. placentodes* strain (X21214) collected from Tibet. The strain was confirmed as *P. placentodes* based on 96% ITS sequence similarity and consistent morphological characteristics. Growth-parameter experiments showed that the species grows optimally with fructose and sucrose as carbon, yeast powder and beef powder nitrogen sources, respectively, at pH 5.0 and 20 °C, reflecting adaptation to cool, mildly acidic environments. Under optimized substrate conditions, *P. placentodes* completed colonization in 23 days, primordia formation in 10 days, and fruiting in 7 days, achieving a biological efficiency of 11.5%. Nutritional analysis indicated high protein (30.3%) and amino-acid contents (21.5 g/100 g) with low fat (1.0%), underscoring its potential as a nutritious, functional edible mushroom.

The successful domestication of *P. placentodes* lays a scientific foundation for its industrial cultivation and germplasm preservation, thereby reducing reliance on wild harvesting and promoting sustainable utilization. However, as this study is preliminary, employing only a single strain and a specific substrate formula, future work should focus on optimizing substrate composition, evaluating yield stability over multiple production cycles, and scaling up cultivation trials. Subsequent research should also explore bioactive compounds with potential medicinal value, such as polysaccharides and secondary metabolites, and establish scalable cultivation systems suitable for high-altitude or low-temperature regions. These efforts will advance the commercial and medicinal development of *P. placentodes* and contribute to the diversification of edible fungal resources in China.

## Figures and Tables

**Table 1:** Fruiting results of *P. placentodes*.

Strain Name	Full Bag Time (d)	Primordium Occurrence Time (d)	Harvest Time (d)	Average Fresh Weight per Flower (g)	Whether There Is a Second Tide
*P. placentodes*	23	10	7	39.40	Yes

**Table 2:** Nutrient content of *P. placentodes* fruiting bodies and three other common edible mushrooms (*Pleurotus eryngii*, *Flammulina velutipes* and *Pleurotus ostreatus*).

Nutrient Content	Content (g/100 g)			
	*P. placentodes*	*P. eryngii* [15]	*F. velutipes* [15]	*P. ostreatus* [15]
Moisture (g/100 g) (fresh)	91.45	90.26	89.32	90.89
Moisture (g/100 g) (dry)	16.0	4.62	4.45	6.75
Ash (g/100 g)	6.5	4.90	7.23	9.47
Fat (g/100 g)	1.0	5.13	5.51	5.54
Protein (g/100 g)	30.30	16.39	20.64	21.24
Soluble sugar (g/100 g)	6.60	1.50	1.50	4.72
Dietary fiber (g/100 g)	29.2	34.03	37.27	41.85
Na (mg/100 g)	8.77	-	-	-

Note: - Indicates not detected or traced (lower than the detection limit of the currently used detection method). The nutrient content was measured in the dry weight .

**Table 3:** Analysis of amino acid composition in the fruiting bodies of *P. placentodes* and three other common edible mushrooms.

Amino Acid Composition	Content (g/100 g)			
	*P. placentodes*	*P. eryngii* [15]	*F. velutipes* [15]	*P. ostreatus* [15]
Asp	2.15	1.83	1.58	1.41
Glu	4.49	2.95	4.41	5.90
Ser	1.21	0.71	0.66	0.55
His *	0.47	0.50	0.60	0.47
Gly	1.02	0.97	0.94	0.80
Thr *	1.15	0.82	0.77	0.70
Arg	1.48	1.14	0.85	0.68
Ala	1.52	1.25	1.21	1.23
Tyr	0.58	0.44	0.63	0.08
Cys	-	0.06	0.05	0.05
Val *	1.23	1.57	1.54	1.24
Met *	0.75	0.23	0.21	0.17
Phe *	0.93	0.72	1.01	0.57
Ile *	0.7	0.83	0.76	0.62
Leu *	1.39	1.19	1.14	0.91
Lys *	1.34	1.11	1.20	0.70
Pro	1.05	0.29	0.27	0.32
Total amino acids (TAA)	21.5	16.59	17.82	16.38
Essential amino acids (EAA)	7.96	6.97	7.23	5.38
Non-essential amino acids (NEAA)	13.54	9.62	10.59	11.0
EAA/TAA	0.37	0.42	0.41	0.33
EAA/NEAA	0.59	0.72	0.68	0.49

Note: * denotes essential amino acids; - Indicates not detected or traced (lower than the detection limit of the currently used detection method). The amino acid composition was measured in the dry weight.

## Data Availability

The original contributions presented in the study are included in the article, further inquiries can be directed to the corresponding author.
